# Hypospadias, all there is to know

**DOI:** 10.1007/s00431-017-2864-5

**Published:** 2017-02-11

**Authors:** H. J. R. van der Horst, L. L. de Wall

**Affiliations:** 1 Department of Urology, VUmc, De Boelelaan 1117, P.O. Box 7057, 1007 MB Amsterdam, The Netherlands; 2 Department of Urology, Radboudumc, Geert Grooteplein 10, P.O. Box 9101, 6500 HB Nijmegen, The Netherlands

**Keywords:** Hypospadias, Disorders of sex development, Timing of treatment, Conservative management, Tissue engineering, Referral, Algorithm

## Abstract

Hypospadias is one of the most common congenital anomalies in men. The condition is typically characterized by proximal displacement of the urethral opening, penile curvature, and a ventrally deficient hooded foreskin. In about 70%, the urethral meatus is located distally on the penile shaft; this is considered a mild form that is not associated with other urogenital deformities. The remaining 30% are proximal and often more complex. In these cases, endocrinological evaluation is advised to exclude disorders of sexual differentiation, especially in case of concomitant unilateral or bilateral undescended testis. Although the etiology of hypospadias is largely unknown, many hypotheses exist about genetic predisposition and hormonal influences. The goal of hypospadias repair is to achieve cosmetic and functional normality, and currently, surgery is recommended between 6 and 18 months of age. Hypospadias can be corrected at any age with comparable complication risk, functional, and cosmetic outcome; however, the optimal age of repair remains conclusive. Although long-term overall outcome concerning cosmetic appearance and sexual function is fairly good, after correction, men may more often be inhibited in seeking sexual contact. Moreover, lower urinary tract symptoms occur twice as often in patients undergoing hypospadias repair and can still occur many years after the initial repair.

*Conclusion*: This study explores the most recent insights into the management of hypospadias.

**Table:** 

**What is Known:**
• *Guidelines advise referral for treatment between 6 and 18 months of age*. • *Cosmetic outcome is considered satisfactory in over 70% of all patients*.
**What is New:**
• *Long-term complications include urinary tract symptoms and sexual and cosmetic issues*. • *New developments allow a more individualized approach, hopefully leading to less complications and more patient satisfaction*.

## Introduction

**Fig. 1:**
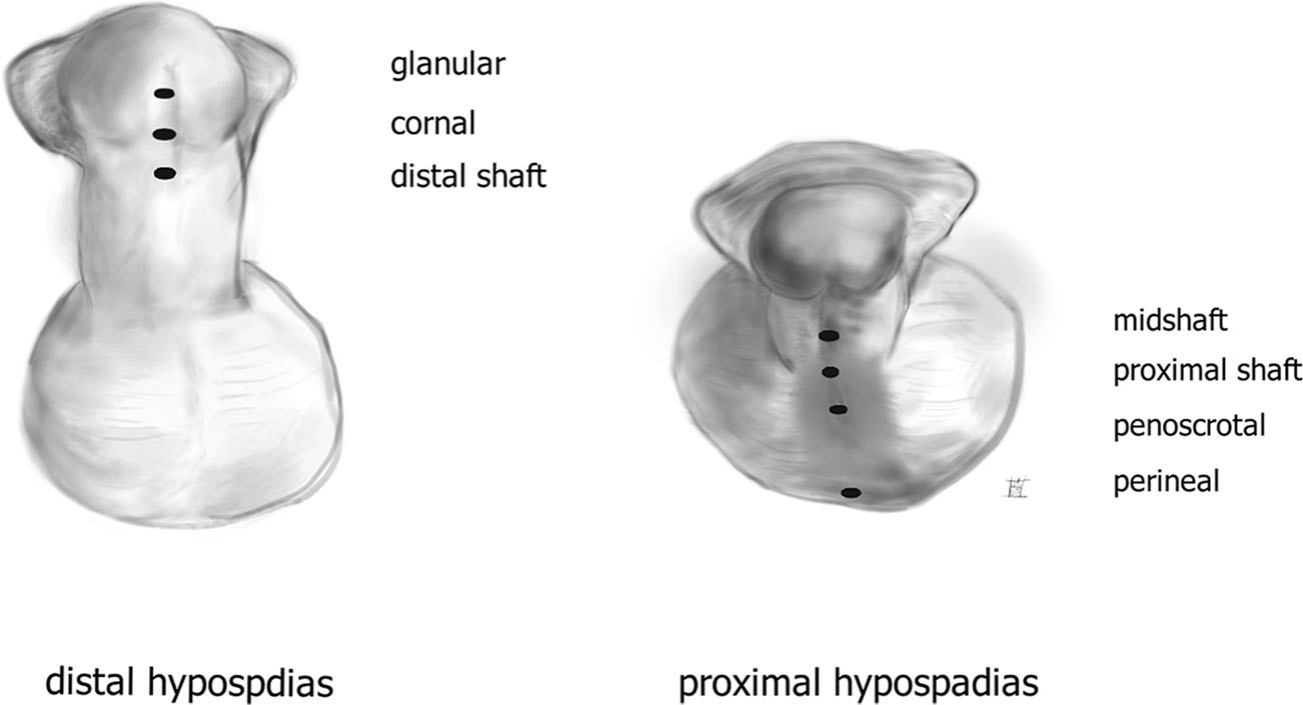
Classification of hypospadias based on preoperative position of the meatus

In newborn males, hypospadias is the second most common congenital anomaly after undescended testis [8]. Due to incomplete closure of the penile structures during embryogenesis, the urethral opening is displaced along the ventral side of the penis [8]. Hypospadias is often classified in posterior, penile, and anterior according to the preoperative meatal position [20]. Duckett proposed the most commonly used classification; i.e., nearly 70% of hypospadias are either glanular or distally located on the penis and are considered a mild form, whereas the remainder is more severe and complex [20] (Fig. 1).

The criteria used to define and evaluate hypospadias are not well described. Meatal position alone is generally accepted to be a very crude way to classify severity of hypospadias and does not take into account the amount of tissue dysplasia. Factors such as size of the penis, size of glans and urethral plate, level of division of the corpus spongiosum, presence of a curvature, and anomalies and position of the scrotum also have a significant influence on the outcome of surgical correction. Therefore, a definite classification can only be completed during surgery [45].

This non-systematic review presents an overview and discusses some controversies related to this field. As pediatricians are often the first to detect hypospadias, they play an important role in consulting parents before referral for surgical intervention. This review aims to provide a useful guide with updated information for optimal initial counseling. For this, a search was made in English language literature using a combination of keywords (including hypospadias, embryology, epidemiology, etiology, diagnostics, treatment, long-term outcome, and future perspectives).

## Embryology

The first, hormone-independent stage of genital development consists of forming a urethral plate in the midline of the genital tubercle. This takes place during weeks 8 and 12 of gestation in both male and female fetuses. During the second stage, between 11 and 16 weeks of gestation, the genital tubercle elongates under the influence of fetal testicular androgens. The urethral plate elongates into a groove towards the tip of the phallus. Fusion of the labioscrotal folds in the midline forms the scrotum, and fusion of the urethral folds adjacent to the urethral plate results in creation of the penile urethra. Eventually, the glans of the penis and the foreskin close in the midline [7].

## Epidemiology

The prevalence of hypospadias in Europe is approximately 18.6 per 10,000 births. Despite previously reported increasing and decreasing temporal trends, hypospadias registered between 2001 and 2010 in 23 EUROCAT registries revealed a stable number [6]. The prevalence is highest in North America, 34.2 per 10,000 births (range 6–129.8), and lowest in Asia, i.e., 0.6–69 per 10,000 births. Despite more than 90,000,000 screened births, true worldwide prevalence and trends remain difficult to estimate due to many methodological factors [47].

Giving the prevalence, hypospadias can be a substantial burden on health care resources [8]. Several surgeries might be required, especially in the severe cases, due to a high risk of complications. Additionally, a significant percentage of patients suffer from cosmetic or functional difficulties [3].

## Etiology

Many hypotheses have been proposed concerning the etiology of hypospadias, including genetic predisposition, inadequate hormonal stimulation prenatally, maternal-placental factors, and environmental influences. Thus, it seems possible that the etiology of hypospadias is multifactorial [5, 42].

## Genetic predisposition

Familial clustering is seen in hypospadias, with 7% of cases having affected first-, second-, or third-degree relatives. Familial occurrence seems more common for anterior and middle forms than for posterior types. The chance that a male sibling of an affected boy will have a hypospadias is 9–17%. Hypospadias are equally transmitted through the maternal and paternal sides of the family, with an estimated heritability of 57–77% [50]. In only 30% of hypospadias is a clear genetic cause found [40]. Hypospadias have been described in over 200 syndromes. The two most well-known are the Wilms’ tumor, aniridia, genitourinary malformations, and mental retardation (WAGR) and the Denys-Drash syndrome (genitourinary malformations and susceptibility to Wilms’ tumor) [7].

## Maternal and other possible influences

Epidemiological studies found an increased incidence of hypospadias in children with small gestational age and in monochorionic twins [22, 23]. Furthermore, severe hypospadias are associated with maternal hypertension, oligohydramnios, and premature delivery, suggesting that underlying placental insufficiency may be an important factor, possibly through inadequate provision of hCG to the fetus [26]. Some studies found a fivefold increased risk of hypospadias for a male newborn conceived by IVF/ICSI. While these conception methods are directly associated with low birth weight and prematurity, and both known to increase the occurrence of hypospadias, controversy still exists as to whether or not this is an indirect effect [4, 43].

## Hormonal and environmental influences

Most hypospadias occur as an isolated condition, but associated anomalies include uni-bilateral cryptorchidism and micropenis [25]. The occurrence of these co-morbidities suggests a deficiency of hormonal influences during embryogenesis. Androgens and estrogens both play a critical role in genital development, and in case of disbalance, different entities can be seen within the spectrum of congenital penile anomalies like hypospadias, micropenis, and ambiguous genitalia [25]. One clinical finding to support this theory is a reduced anogenital distance in boys with hypospadias as a result of disruption of prenatal androgen exposure [48]. Other studies emphasize the potential effect of so-called environmental endocrine-disrupting chemicals on the development of hypospadias. This is mainly based on animal studies, in which maternal exposure to synthetic estrogens induced hypospadias in murine models. However, because of the considerable differences between species, it remains debatable whether it has any substantial influence in humans [50].

Another important hypothesis postulates some male reproductive disorders (cryptorchidism, hypospadias, male subfertility, and testicular cancer) to be interlinked and originated from a disturbed testicular development. This is known as the testicular dysgenesis syndrome [44]. Such impairment could be caused by the influence of all the etiological factors mentioned above.

## Diagnostic evaluation

Hypospadias is generally defined as the combination of three anatomic anomalies of the penis, which are an abnormal ventral opening of the urethral orifice, ventral curvature of the penis, and abnormal distribution of the foreskin around the glans with a ventrally deficient hooded foreskin [27]. Ventral curvature and lack of circular ventral union of the prepuce are not always present. Special variations of hypospadias are the so-called hypospadias sine hypospadias and the megameatus intact prepuce (MIP). The first is characterized by a ventral curvature of the penis and a normal position of the meatus with a distorted foreskin. The latter is characterized by a coronal lying meatus adjacent to a non-closed glans with a very wide open navicular fossa and a normal developed circular prepuce [21, 32].

## Endocrinological evaluation

In case of concomitant unilateral or bilateral undescended testis, one should always be aware of a disorder of sex development (DSD). The incidence of DSD in patients with simple distal hypospadias is similar to the incidence in the general population but is increased in proximal or complex hypospadias [28]. In these cases, referral to an endocrinologist for a full genetic and hormonal evaluation is warranted.

## Ultrasonography and endoscopy

In proximal and complex hypospadias, further diagnostic evaluation is advised, such as ultrasonography of the urinary tract and internal genital organs to detect other nephro-urological malformations [28]. A Müllerian remnant (utricular cyst or dilated utriculus) is seen in 11–14% of all hypospadias and up to 50% of perineal hypospadias [36]. Most of these can be visualized by ultrasound. Undetected Müllarian remnants can cause urethral obstruction or urinary tract infections after hypospadias repair. Endoscopic examination of the urethra at the time of surgery can exclude the presence of urethral anomalies not detected by ultrasound [28].

## Controversies in treatment

The main goal for hypospadias repair is to achieve both cosmetic and functional normalities. Reasons for treating hypospadias include spraying of urinary stream, inability to urinate in standing position, curvature leading to difficulties during intercourse, fertility issues because of difficulty with sperm deposition, and decreased satisfaction with genital appearance [37]. Current guidelines consider optimal age for hypospadias repair somewhere between 6 and 18 months, depending on the severity and the need for multiple procedures [37]. Anesthetic risks, age-dependent tissue dimensions, and psychological effect of genital surgery all have certain effects [28]. In the last decennia, alarming results have been published concerning anesthetic-induced neurodegeneration on the developing central nervous system in rats [31]. However, methodological issues make it questionable whether these findings are of any importance in humans [31]. A recent randomized controlled trial showed no difference in neurodevelopment outcome between children operated in awake regional and in general anesthesia [15].

Penile biometrical parameters, like a small glans width and narrow urethral plate, are some of the anatomical factors associated with increased postoperative complications and form a technical challenge [11, 14]. However, penile size in general is rarely considered a limiting factor concerning the optimal time of hypospadias repair, as only moderate penile growth occurs in the first few years of life. Therefore, delay of surgery does not seem to be of any advantage [28]. To increase anatomical proportions, some surgeons advocate testosterone supplement in case of a microphallus, which is defined as a penile length below the third percentile [54]. Data on the effects of testosterone supplement prior to hypospadias repair are both limited and of poor quality. In a systematic review by Wright el al., a trend was seen towards an increased risk of complications of preoperative intramuscular testosterone in patients with severe hypospadias; nowadays, this treatment is less frequently used [54].

Adolescents who did not recall the surgery were more likely to have a positive body image and be satisfied with their overall body appearance than those who did [12]. Because genital awareness is known to start at the age of 18 months, surgery and hospitalization are less attractive in this age group [28]. These findings apply for surgery early in life to minimize the psychological burden.

Some studies suggest that initial hypospadias repair at a later stage in life could be associated with more postoperative complications [19, 30]. Postoperative factors, like the amount of urethral secretions and nightly erections, might have some influence, possibly leading to more infections, hematoma, and wound breakdown [19]. However, other studies found no association between age of initial hypospadias repair and number of complications [10, 46].

These controversial findings concerning possible anesthetic risks, psychological impact and postoperative complications, have led to discussion as to whether or not surgery should be delayed until the child is able to meaningfully participate in the decision-making process [12]. As most of these studies are based on retrospectively gathered data of a single surgeon/center, additional studies are definitely needed. One such initiative is “The Dutch hypospadias database,” which contains prospectively collected data from all hypospadias repairs performed in the Netherlands from 2010 onwards. Data from this database and further European implementation might provide better insight into various questions, including the optimal time frame for hypospadias repair.

## Long-term outcomes

While the majority of current hypospadias research is based on observational reports, the literature lacks standardization of techniques for hypospadias repair and uniform definitions of complications and outcome assessment [9]. Many different questionnaires (each with their own advantages/disadvantages) have been developed to evaluate the outcome after hypospadias repair. Some frequently used are the (Pediatric) Penile Perception Score (PPPS), the Hypospadias Objective Scoring System (HOSE), the Pediatric Quality of Life Inventory (PedsQl), and the Hypospadias Objective Penile Evaluation Score (HOPE) [24, 49, 51, 52].

**Fig. 2:**
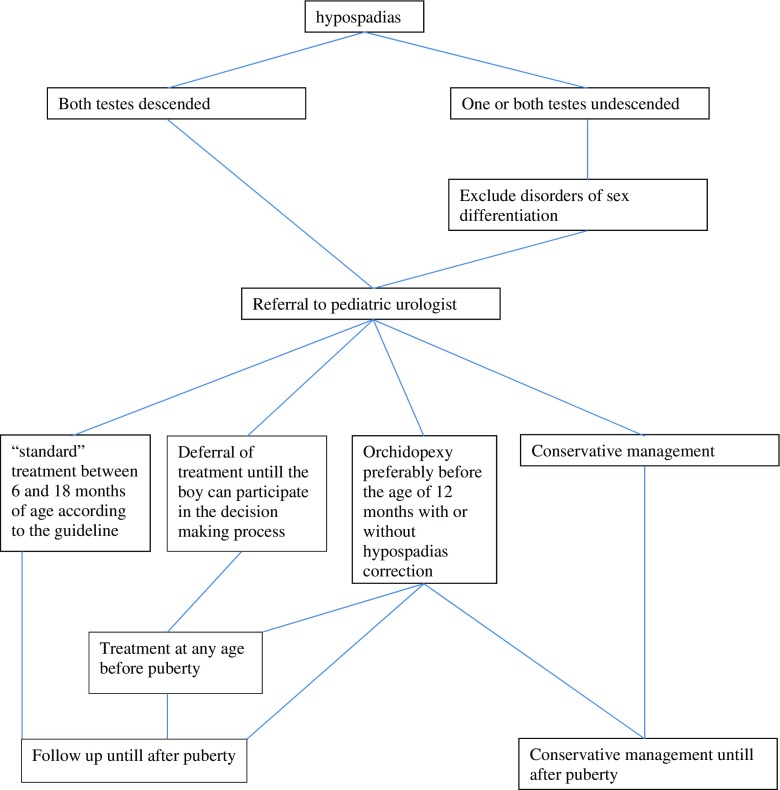
Proposed algorithm for referral and treatment of hypospadias

Currently, no standardized questionnaires are available for the evaluation of psychosexual function after hypospadias repair [17]. Functional outcome is mainly assessed by uroflowmetry and postvoid residual measurements (Fig. 2).

To increase the quality of research in this field and to enable better comparison between different observational studies, standardization in the reporting of cosmetic and functional outcome using objective, reproducible, and validated tools are essential and of utmost importance [9].

## Long-term cosmetic and sexual outcomes

Overall, cosmetic outcome is considered satisfactory in more than 70% of all patients after hypospadias repair [39]. The worst results (from self-reported questionnaires) are in those patients treated for proximal and complex hypospadias; in this group, more than 50% were dissatisfied with the appearance of their penis [39]. Few studies have addressed the perception of untreated hypospadias by the patients or by others. Moreover, the few available studies show conflicting results concerning function and cosmetic appearance in men with untreated hypospadias. Some report worse outcomes compared to men without hypospadias, while others report an overall satisfaction rate of 95% [18, 41]. As expected, mild untreated hypospadias had fewer adverse outcomes than severe hypospadias [18].

In general, sexual function in men with corrected hypospadias was satisfactory in more than 80% [39]. However, these patients are more often inhibited in seeking sexual contact or are more often afraid of being ridiculed by others because of the appearance of their penis [33, 39]. A study performed by Ruppen-Greeff et al. revealed that laypersons do not notice a difference between corrected distal types of hypospadias and otherwise circumcised genitals. Furthermore, women considered the position and shape of the meatus as the least important penile aspect [38].

## Long-term functional outcomes

Lower urinary tract symptoms were twice as common in patients who had undergone hypospadias repair than in controls [39]. An obstructive urinary flow pattern is frequently seen after tubularized incised plate (TIP) urethroplasty, which might be caused by abnormal elastic qualities of the created tube [53]. After proximal hypospadias repair, almost 39% of the patients reported voiding dysfunction, mainly hesitancy and spraying [39]. Objective parameters (e.g., maximal flow rate) were found to be less in severe hypospadias, but only slight differences were found in patients after mild hypospadias repair and controls [35]. Differences in tissue surrounding the neo-urethra (like scar tissue) might explain variances in compliance, resulting in variances in maximal flow rate [35]. Interestingly, normalization of previous abnormal voiding patterns also seems possible. In a study by Andersson et al., normalization of urinary flowmetry at puberty was seen in 95% of children after TIP repair for hypospadias in childhood [1]. Urinary complications (e.g., meatal stenosis, fistula, or urethral stenosis) can still occur years after initial repair, and long-term follow-up is therefore advised [34].

## Current opinion: future perspectives

Despite more than 250 different techniques for hypospadias repair, successful outcome depends mainly on the surgeon’s skills and the availability of appropriate tissue. In case of insufficient tissue, oral buccal mucosal grafts are one of the alternatives [29]. Unique histological characteristics, such as thin lamina propria and thick epithelium, facilitate optimal vascular supply and inosculation [29]. All substituted tissues from other origins (skin, bladder, or buccal mucosa) have their own limitations, which can increase complications like stricture formations and graft failure [16]; furthermore, the amount of tissue harvested can be limited. Alternative sources of tissue have been proposed over the years, such as autologous cell cultures, matrices/scaffolds, and cell-seeded scaffolds [2, 16]. Different progenitor cells have been used, harvested from either urine or adipose tissue. Thus far, the best results were obtained using in vitro expansion of cells from bladder washings, oral cavity, or tissue biopsies (bladder) [2, 16]. Two strategies are available for urethral reconstruction using tissue engineering, the acellular matrix bioscaffold and the cell-seeded bioscaffold model. Biomaterials in genitourinary tissue engineering are either naturally derived (collagen, alginate, acellular tissue matrices like bladder submucosa) or synthetic polymers (polyglycolic acid, polyactid acid, polylactic-co-glycolic acid). The latter can be produced on a large scale but have the potential disadvantage of host versus graft reactions [16]. Successful urethral repair was possible with acellular matrices in both rabbits and patients with failed hypospadias reconstruction as inlay urethral repairs [16]. However, tubularized urethral repairs with acellular matrices resulted in graft contracture and stricture formation [16]. Clinical experience in this area is still relatively scarce, and further research is needed before tissue-engineered urethral repair will become daily practice. Meanwhile, due to the above-mentioned characteristics, for over 20 years, oral mucosa is considered by most surgeons as the best and therefore the primary source of alternative tissue in complex hypospadias [13].

## Conclusion

Hypospadias is a common condition with an unknown etiology, with considerable variety in its presentation and severity. The goal for hypospadias repair is to normalize function and cosmetics. Generally, hypospadias is corrected between 6 and 18 months of age but (concerning results and complications) can be performed at any age. Optimal age for surgical intervention is still debated and influenced by anesthetic risks, tissue dimensions at different ages, postoperative complications, and psychosocial impact. Both functional and cosmetic long-term outcomes are generally acceptable but are still inferior to the situation in men without hypospadias.

## Abbreviations

DSD: Disorder of sex development
hCG: Human chorion gonadotropin
MIP: Megameatus intact prepuce form of hypospadias
WAGR syndrome: Wilms’ tumor, aniridia, genitourinary malformations, and mental retardation
PPPS: Pediatric Penile Perception Score
HOSE: Hypospadias Objective Scoring System
PedsQl: Pediatric Quality of Life Inventory
HOPE: Hypospadias Objective Penile Evaluation
TIP: Tubularized incised plate urethroplasty
